# Combating substance misuse: competences and preparation of special education department students

**DOI:** 10.1186/s13011-016-0086-x

**Published:** 2017-01-14

**Authors:** Eman Al-Zboon

**Affiliations:** Special Education Department, Queen Rania Faculty for Childhood, Hashemite University, Zarqa, 13115 Jordan

**Keywords:** Substance misuse, Student with disabilities, Pre-service teachers

## Abstract

**Background:**

This study examines Jordanian special education department students’ competences and preparation relating to combating substance misuse (SM).

**Method:**

Thematic analysis was performed on data from interviews with 150 students.

**Results:**

Some participants denied the possibility of addiction among students with disabilities, and presented negative attitudes toward their role in combating SM. In general, the participants displayed low levels of professionalism relating to combating SM, and the results revealed that they felt that their preparation programme had been inadequate and they desired more courses that related to combating SM.

**Conclusions:**

These results suggest that Jordanian Universities should emphasise the role of teachers in a preventive approach to SM.

## Background

Substance misuse (SM) is one of the most serious human health problems worldwide [1]. For some groups of students with disabilities (SWDs), levels of SM may be higher, as they encounter a greater number of risk factors [2], including medication and health problems, societal enabling, a lack of identification of potential problems, and a lack of accessible and appropriate prevention and treatment services [3]. Furthermore, stresses faced by SWDs may lead to the higher possibility of nicotine addiction, alcohol addiction, or drug addiction [4].

Schools should be alert to the fact that SM can be higher in some groups of SWDs [2]. However, the type of disability strongly conditions the actual prevalence of substance use and substance use disorders. Westermeyer, Kempt and Nugent [5] reported that persons with intellectual disabilities and SM disorder comprised 6.2% of 642 individuals receiving treatment for SM at two clinics. However, available literature suggests that relatively little epidemiological work has been done and SM is a hidden problem among the people with disabilities (PWDs) (e.g., [6]).

Teachers hold the central role in education for SM prevention, and teacher training is an important component of any SM prevention programme. Education for SM prevention is more effective when teachers receive formal training and have access to ongoing advice and support [7], although their own beliefs and views on SM must be thoroughly understood before any initiatives take place. Training teachers in SM prevention education enhances the impact and sustainability of SM prevention programmes, and this is especially critical if they are to be teachers of SWDs. Appropriate pre-service and continuing education related to SM and disability for practitioners has the potential to lead to improved psychosocial outcomes for individuals with disabilities [8].

Jordan is a small country that suffers from both political and economic problems, accentuated over recent years following the ‘Arab Spring’ and the influx of refugees from conflict countries [9]. This situation is reflected within the health, rehabilitation and education services, and notably in services designed for SWDs as they have a lower priority. Moreover, Jordan has been the crossroads of drugs between the East/West and North/South routes of commerce in the Middle East. Jordan also is in close proximity to economically disadvantaged countries that produce drugs. Drug use could be expanded because of the migration of the rural poor to the larger cities, a decline in illegal drug prices, and the recent inflow of refugees from conflict countries [10]. Unfortunately, epidemiological data on the situation on SM among PWDs in Jordan are very scarce.

In Jordan there are many universities that have special education (SE) departments, and the SE teacher preparation programme at Jordanian Universities is introduced at the undergraduate level as a 4-year programme of study. This programme prepares students to be teachers of children with disabilities. The SE teaching programme does not include any courses related to combating SM, although it does include some courses taught by lecturers who specialise in SE and which talk about SM in general, including Child Rights and Laws, Child Abuse, Mental Health Care for Children, and Behavioural and Emotional Disorders. The SE teachers’ programme prepares teachers to deal with SWDs at the undergraduate and graduate levels, and many Jordanian studies have revealed the importance of the reformation process to these programmes (e.g., [11]). However, none of these studies have examined the programmes in relation to combating SM in terms of the teachers’ professional competences and evaluation of the preparation programme.

Therefore, this study aims to examine this topic and fill the knowledge gap, as this knowledge is vital for the decision-makers of these programmes so that they can be altered appropriately and prepare teachers for combating SM. Qualitative research aims to collect rich in-depth data about a topic, in order to detect recurring themes; these can then be used to informand monitor service development and/or subsequent rigorous quantitative research [12, 13].

## Method

### Participants

In this qualitative study, 150 (62 male and 88 female) SE students from Jordanian universities were recruited through their departments, using a purposive sample selection method. Final selection of the sample was made after securing the essential permissions. Participation was voluntary and all the students were required to sign an informed consent form. The group of students selected all met the following criteria: they must be attending the SE departments, from the same cohort of fourth-year Bachelor of SE students, and had finished all their courses and practicum semester. The rationale for choosing students in their fourth years was to ensure that they would have the most experienceof the SE programme at their university. The large sample size for this qualitative study raises the concern that breadth rather than depth was prioritised, but the rationale was the belief that the opportunity to potentially elicit the opinions of all or most of the final year students was worth the attempt. In the field, the interviews ranged from 30 to 45 min. We were able to provide time for each participant to elaborate on their answers, giving depth to the interviews.

The study was conducted according to ethical principles and received ethical approval from the Institutional Review Board (IRB) at Hashemite University.

### Data collection

This study utilised qualitative research in order to delve into SE students’ perspectives of combating SM. Semi-structured interviews were used based on Kvales’ stages [14]. The interviews provided a wealth of information on how teachers perceive this topic and participants shared a lived experience. The interview schedule designed by the researcher was used to guide the interviews, and a total of five open-ended questions (main questions) were developed which enquired about the possibility of SM among SWDs; the teacher’s role in combating SM among SWDs; their perception of how well prepared they felt to deal with SM; their evaluation of their preparation programme related to providing courses which best prepare them to deal with SM; and services provided in their universities.

The main questions were followed up within the interview, with further inquiries about the answers given. Probing questions were used to clarify, such as ‘Please tell me more.’ ‘Please give me an example.’

### Data analysis

The interviews were tape-recorded with the consent of the participants, and in order to analyse the data each interview was transcribed verbatim. To analyse the data, thematic analysis was employed, as described by Holloway and Todres [15]. This analysis includes seeking patterns, inspecting emergent themes, identifying interrelating themes, and providing quotes from the raw data to support the themes. To confirm the accuracy of the identification of the main themes and to check the credibility of the analysis, the researcher and research assistants read and coded the data independently. Coding was checked for substantive significance [16]. In this stage, each segment of data was marked with a code – a word or a short phrase that symbolically represented and captured participants’ perceptions.

## Results

### Possibility of addiction among SWDs

The interview analysis revealed that 88% of participants denied the possibility of addiction among SWDs, and mentioned the following reasons: family overprotection; limitations placed on them related to movement and mobility within the community; and their disability. However, 12% of the participants did report the possibility of addiction among SWDs and noted the following reasons: addiction is possible for any individual; environmental circumstances; stress; self-confirmation to community; family model; feelings of deficiency; to draw attention; exploitation by others; escape from reality; a lack of self-confidence; to forget pain; the negative views of other people towards SWDs; ridicule from others; calming; psychological issues; lack of cognition; parents being accustomed to behavioural problems.

Some typical responses were:

Nahid: ‘*SWDs are human beings who could be addicted. We should have a realistic view towards them.’*

Rida: ‘*The first reason for addiction among them, from my point of view, is the community attitudes and negative views of other people towards SWDs’.*

### Teachers’ perceptions related to their role in combating substance misuse

When teachers were asked about their role in combating SM among SWDs, one quarter revealed negative attitudes towards their role in combating SM; they considered this not to be part of their role and that there were other persons to deal with SM, including counsellors (*n* = 20), and the anti-narcotics department (*n* = 15). Twenty percent of participants noted that the school staff responsible for combating SM were the teachers and mentioned the following procedures for combating SM: improving students’ awareness related to the negative effects of addiction; inviting professional visitors to talk about addiction; telling stories related to the effects of addiction; raising religious awareness; training courses; improving family involvement, brochures; art and  drama; follow-up appointments for students’ medication; behaviour monitoring; spending time under taking useful activities; seminars; videos; and the referral of students at risk of SM to professionals.

### Teachers’ perceptions regarding their competencies and preparation programme

Ninety-three percent of the participants thought that they had low levels of competences relating to combating SM. When asked about which courses in university had focused on SM and improved their ability to combat SM, the participants listed: Health Education and First Aid, Military Science, Child Rights and Laws, Child Abuse, Mental Health Care for Children, Behavioural and Emotional Disorders, Islamic Education, Arabic Language, and Human Rights.

The results from the study revealed that the participants felt that their preparation programme had been inadequate and they desired further courses related to combating SM. All the participants reported the teaching materials provided had not given them enough information, and all the information they had received related to SM was provided in a single lecture, which had only focused on SM, its definition and different types. None of the courses had discussed the role of teachers and schools or ways to combat SM. Samia reflected on her own personal experience, saying: *‘I attended some courses which talked about SM in general. Unfortunately, none of these course talked about teachers’ roles in combating SM among SWDs.’*

The data analysis revealed that 87% of participants reported that they had not participated in any university activities related to combatting SM. However, 18% of participants reported that they had attended seminars and Ahlam shared her experience: ‘*I don’t think that our preparation programme prepared us for combating SM. I took one course entitled ‘Health Education’ where the lecturer talked about SM as an aside.’*

## Discussion

This study provides evidence that pre-service SE teachers have low levels of professionalism relating to combating SM. However, these findings are not surprising, since the topic of integrated SM education in pre-service teacher preparation programmes is viewed as a recent practice globally. This topic is a new trend in teacher preparation programmes and the results reflect the worldwide call highlighting the importance of increasing levels of professionalism among teachers relating to combating SM [7]. One of the most significant ways to improve teachers’ level of professionalism is through pre-service teacher education [17]. This result emphasises this, as the preparation programmes at Jordanian universities are evidently not providing pre-service training for all graduate students in this area, therefore the Ministry of Education (MOE) should provide in-service training to fill this gap in teacher training knowledge.

This study showed that 12% participants were aware of the possibility of addiction among SWDs, and this is in line with many studies which have found that addiction among SWDs is common [18–20]. A recent study shows a 12.2% lifetime prevalence estimate of medication therapy for Attention-Deficit/Hyperactivity Disorder [21]; and as Eberstadt [22] asserts, it seems that schools are now stressing not three but four R’s; Reading, Writing, ‘Rithmetic, and Ritalin.

A very high number of participants in this study held incorrect perceptions regarding two vital topics in combating SM among SWDs. The first was denying the possibility of addiction amongst SWDs, as this result is inconsistent with research that reports SM in SWDs [5, 18, 19]. SM maybe higher in some groups of SWDs as they face risk factors that can lead to SM [2]. This incorrect perception can be accounted for in two ways: first, through the community beliefs, social stigma and dehumanising perceptions that prevail in Arab countries regarding SWDs [23, 24], meaning that the possibility of addiction among SWDs is perceived as low. Another reason may be the pre-service teachers’ lack of experience of SWDs, as these Jordanian teachers had undertaken only one practical semester in the field. These participants had potentially only encountered one category of SWDs and they had not had sufficient time to explore addiction among SWDs.

The second incorrect perception was participants’ negative attitudes toward their role in combating SM. Most of the teachers considered that combating SM was not part of their role, and thought that there were other professionals who should deal with it. This perception is considered as one of the main constraints to the introduction of prevention programmes against SM within schools for SWDs. The study participants were future teachers, and many studies have indicated the importance of teachers’ perceptions in improving services [25]. Graham, Phelps, Maddison, and Fitzgerald [26] reported that teachers’ perceptions played a key role in the significance they placed on schools enhancing students’ mental health. Furthermore, these authors stated that close attention should be paid to the beliefs and attitudes of teachers in relation to students’ mental health, because these variables may predict their confidence and skills in supporting students’ well-being. Future studies should look to provide logical explanations for these perceptions.

The participants emphasised the role of teachers and counsellors in combating SM, and relationships with teachers and counsellors are among the most important and formative ones for students [27]. School counsellors address students’ personal/social needs by providing services through prevention education, responsive services, and in collaboration with community members [28]. Recent literature has focused on social responsibility in combating the problems of students of any age, and teachers are one of the vital stakeholders in social responsibility [7]. In addition, recent educational literature has indicated that the teacher’s role is that of an educator not an information transit. Teachers need to encourage the development of students’ personalities and counsel them against problems, such as SM, through the curriculum and extra-curricular activities, for example support groups, and sport and cultural activities [29].

None of the participants mentioned the role of other school staff. It is crucial that all other staff members participate and demonstrate their commitment, rather than only those who perceive themselves to have a particular role in students’ health or welfare. All teachers have a duty of care to recognise and refer young people who are at particular risk of mental health problems, disorders or self-harm [30], although education authorities should not accept sole responsibility for changing student health behaviours, including reducing SM [7].

The results from the study revealed that the participants felt that their preparation programme had been inadequate and they desired more courses relating to combating SM. However, this result is inconsistent with the recent trend that emphasises drug abuse prevention education during teacher training, and its inclusion has been found to enhance the impact and sustainability of SM prevention programmes [7].

Most of the participants reported that they did not participate in any university activities or receive any services related to combating SM. This result is in line with the findings of a previous study [31], which indicated that few services related to SM are provided in universities. Most university counselling or health service centres are insufficiently equipped to support students in recovery or to assist students with SM. However, some universities are starting to become actively engaged in providing comprehensive policies and services in order to tackle SM issues [32]. This is more critical for teachers who hold a central role in providing education for drug abuse prevention through the provision of access to professional development via workshops, seminars, conferences and networking, and through providing them with basic counselling skills [7].

## Limitations

This study has some limitations which deserve attention when interpreting the results. A primary issue is the representativeness of the sample, as the participants were purposefully chosen and therefore the findings are not necessarily representative for the larger population of pre-service SE teachers in other universities. A second important limitation relates to the research methodology, which depended on interviews alone. Future studies should use other methods to investigate this topic, such as document analysis to examine SE programmes and scrutiny of syllabi to provide a more accurate programme evaluation and to enable comparisons with universal practices.

## Conclusions

The present study revealed that SE students at Jordanian universities felt that their preparation programme as SE teachers were inadequate, and lacking concepts for combating SM among SWDs. From the results of this study it is apparent that there is an urgent need for Jordanian policy-makers and teacher trainers to consider this topic in order to improve teachers’ competences in combating SM. This could be achieved by including more courses related to the role of teachers in combating SM during teacher training. Further research is recommended to explore combating SM in other groups of teachers and professionals working with SWDs in both pre-service and in-service settings, and quantitative research needs to be conducted to achieve a better overall picture regarding combating SM among pre-service teachers in more settings.

## Abbreviations

SE: Special education
SM: Substance misuse
SWDs: Students with disabilities
